# Investigating the impact of COVID-19 lockdowns on fragility fracture risk and bone mineral density in a large observational cohort: a cross-sectional study

**DOI:** 10.1093/rap/rkae115

**Published:** 2024-09-18

**Authors:** Hamzah Amin, Muhammed Aqib Khan, Marwan Bukhari

**Affiliations:** Lancaster University Medical School, Faculty of Health and Medicine, Lancaster University, Lancaster, UK; Department of Rheumatology, University Hospitals of Morecambe Bay NHS Foundation Trust, Lancaster, UK; Lancaster University Medical School, Faculty of Health and Medicine, Lancaster University, Lancaster, UK; Department of Rheumatology, University Hospitals of Morecambe Bay NHS Foundation Trust, Lancaster, UK

**Keywords:** COVID-19, lockdown, bone density, fragility fracture

## Abstract

**Objectives:**

Severe acute respiratory syndrome coronavirus 2 (SARS-COV-2 or COVID-19) led to lockdowns predisposing people to sedentary lifestyles and unhealthy behaviours which may have affected bone mineral density (BMD) and fragility fracture risk. However, limited studies describe such an association. We aimed to investigate how COVID-19 lockdowns has affected BMD and fragility fractures in a large cohort.

**Methods:**

Patients were referred to our DXA scanner from 2004 to 2024 and were subsequently categorized as pre- or post-March 23, 2020 (pre- and post-COVID-19) to allow analysis between the groups. Demographic, BMD and compositional data were compared between the two populations. A multivariate logistic regression modelled the odds of reporting a fracture including hip and non-hip fracture. A multiple linear regression was used to model how the lockdown has affected bone density. All analyses were adjusted for confounders.

**Results:**

Of 43 799 referrals, 6564 were post-COVID-19. Post-COVID-19 patients had higher non-hip fracture rates (42.0% vs 39.8%), were 3 kg heavier, and had lower left femoral T-scores. Patients referred post-COVID-19 had a statistically significant reduction of −0.23 to their T-score after adjusting for confounders as well as increased risk of getting diagnosed with osteoporosis [odds ratio (OR) 1.49, 95% CI 1.40–1.59]. Patients referred after the pandemic had a reduced odds of any fracture (OR 0.83, 95% CI 0.77–0.88), hip (OR 0.74, 95% CI 0.62–0.88) and non-hip fracture (OR 0.78, 95% CI 0.73–0.83).

**Conclusion:**

COVID-19 lockdowns may have negatively affected bone; however, this has not translated to an increased fracture risk in our study. Further research is needed with prospective cohorts to corroborate this risk.

Key messages

- Patients referred after COVID-19 lockdowns had reduced bone density compared to those referred before the lockdown.
- Patients referred after the lockdown had a reduced odds of reporting a fragility fracture.
- Long-term impacts of lockdowns on fracture risk need to be explored further with prospective data.

## Introduction

The UK entered lockdown on 23 March 2020, due to the rapid spread of a novel respiratory virus called Severe acute respiratory syndrome coronavirus 2 (SARS-CoV-2 or COVID-19). The first full lockdown lasted from late March to June 2020, with subsequent varying degrees of lockdown until July 2021. While the primary focus was on curbing virus transmission, the lockdowns may have adversely affected bone health [1] and the risk of fragility fractures.

It has been hypothesized that vulnerable elderly population were particularly susceptible to bone loss during the COVID-19 lockdowns because of physical inactivity and social isolation [2]. Furthermore, compositional changes such as obesity, which saw an increase during the pandemic [3], may have precipitated both bone loss and the risk of fragility fracture [4] as patients changed their dietary behaviours [5]. Social isolation, which particularly affected vulnerable elderly populations [6], may have also been a key driver for bone loss with some laboratory research highlighting social isolation as a novel cause of bone loss [7]. Hence, these multitude of factors may have altered patients’ skeletal health with the long-term effects of lockdowns unknown. However, to our knowledge, no research has conclusively shown that the COVID-19 lockdowns have induced bone loss but rather multiple theories have been suggested as previously stated.

Most data have indicated that there was a lower fracture incidence acutely during COVID-19 lockdowns. This is supported by the office for national statistics (ONS) data [8] as well as French [9] and Greek [10] cohorts internationally. However, ONS data demonstrated a rebound increase in fall and fragility fractures post-lockdown which plateaued at pre lockdown rates [8]. Interestingly though, an increased rate of fragility fracture persisted in patients with premorbid frailty as defined by the hospital wide frailty index [8]. However, ONS data was not adjusted for other known predictors of fracture which ultimately limits the conclusion we can make. Consequently, further research is needed to accurately describe this risk while adjusting for other known predictors of fracture; furthermore, an understanding of why patients may have a differing risk of fracture risk post-lockdown needs to be explored; hence, the rationale of this study. We aimed to investigate how the COVID-19 lockdown has affected fragility fracture risk as well as bone mineral density in a large observational cohort.

## Methods

### Data collection

Patients were referred from primary and secondary care in the Northwest of England to our dual energy X-ray absorptiometry (DXA) scanner between 2004 and 2024. A GE Lunar DXA scanner was used to measure bone mineral density (BMD) and body composition, including body fat percentage. Most patients received a bilateral femoral and lumbar spine (L1–L4) scan, providing fat mass, lean mass, and BMD data for these sites.

Patient demographic details were collected by the technician via a questionnaire at each appointment, including osteoporosis and fragility fracture risk. The technician also measured the patient’s height and weight to calculate BMI. Data were gathered on whether the patient was a current smoker, was taking glucocorticoids (defined as >5 mg for >3 months as per FRAX), or consumed excess alcohol (>3 units per day). Additionally, the patient’s medical history, including rheumatoid arthritis, previous fragility fractures and family history of fractures, was recorded. However, specific dosages of medications like glucocorticoids were not available to us.

Raw data were collected into a Microsoft Access relational database and then extracted pseudo-anonymously for analysis. Data were subsequently imported into STATA V18 for analysis. The timing of the scan was correlated to the initiation of the COVID-19 lockdown, defined as 23 March 2020, when the government first announced a full lockdown. Patients referred after this date (referred to as post-COVID-19) were coded as a one in our dataset, while patients referred prior to this date (pre-COVID-19) were coded as a zero. This coding formed the basis of our analysis.

Full ethical approval for pseudonymized data extraction in the absence of informed consent was obtained from the local ethics committee, National Research Ethics Service (NRES) Committee Northwest Preston (project number 14/NW/1136).

### Data analysis

Baseline characteristics were compared between pre- and post-COVID-19 referred populations. Student’s t-test was used for continuous, normally distributed data, while Pearson’s chi-squared test was used for categorical data, with significance set at *P* < 0.05.

Our primary analysis focused on whether patients were more or less likely to have had a fragility fracture post-COVID-19, after adjusting for key variables including age, biological sex, BMI, current smoking status, current alcohol excess (>3 units/day), current glucocorticoid (GC) therapy, rheumatoid arthritis, family history of fractures, past history of fractures and the left femoral neck T-score. We utilized a multivariate logistic regression model to assess the odds of reporting a fracture after adjustment, stratifying the analysis into any fractures, as well as non-hip and hip fractures specifically.

For the secondary analysis, we wanted to investigate whether any BMD changes had occurred in patients being referred after the pandemic. To do this, we used a multiple linear regression model to assess whether any reduction/increase in the left femoral neck T-score had occurred before versus after the pandemic and if so the effect size as well. Following on from this, we also performed a logistic regression model to evaluate whether patients were more or less likely to have been diagnosed with osteoporosis, osteopenia or normal BMD at each appointment. Osteoporosis, osteopenia and normal BMD were defined using T-score definitions as applied to the left femoral neck. All analyses were adjusted for the previously mentioned confounders.

## Results

Amongst the 43 799 patients referred between 2004 and 2024, 6564 (15.0%) were referred post-COVID-19. The incidence of non-hip fractures was higher amongst all post-COVID-19 referrals (42.0%) compared with pre-COVID-19 referrals (39.8%). However, there was no significant difference in the incidence of hip fractures between the two groups. Gender distribution differed significantly between post-COVID-19 and pre-COVID-19 referrals, with a higher proportion of females referred post-COVID-19 (90.2%) compared with pre-COVID-19 referrals (87.0%). Additionally, the mean age of post-COVID-19 referrals was slightly higher than that of pre-COVID-19 referrals (67.1 vs 65.5 years). Height, weight, BMI and bone mineral density (T-scores) were all significantly different between the two groups, with post-COVID-19 referrals generally having higher BMI and weight but lower BMD. Patients referred post-COVID-19 also had higher levels of adiposity at all sites we analysed. These results can be seen in Table 1.

**Table 1.** Demographic between pre- and post-COVID referred populations

	Post-COVID-19 referred population	Pre-COVID-19 referred population	*P*-value
Number of patients referred	6564 (15.0%)	37 235 (85.0%)	
Non-hip fracture	2754 (42.0%)	13 978 (39.8%)	<0.001
Hip fracture	329 (5.0%)	1752 (4.7%)	0.281
Gender	5921 females	30 559 females	<0.001
	(90.2%)	(87.1%)	
Age	67.1 years	65.5 years	<0.001
	(SD 12.9)	(SD 12.8)	
Height	163.8 cm	162.0 cm	<0.001
	(SD 8.9)	(SD 8.5)	
Weight	74.5 kg	71.1 kg	<0.001
	(SD 18.0)	(SD 16.3)	
Left femoral T-score	−1.2	−0.9	<0.001
	(SD 1.3)	(SD 1.3)	
Right femoral T-score	−1.2	−1.0	<0.001
	(SD 1.3)	(SD 1.3)	
L1–L4 T-score	−0.7	−0.5	<0.001
	(SD 1.8)	(SD 1.7)	
BMI	27.7	27.0	<0.001
	(SD 6.1)	(SD 5.5)	
FPA%	32.4%	31.4%	<0.001
	(SD 11.7)	(SD11.0)	
FPLH%	30.8%	30.5%	<0.001
	(SD 7.8)	(SD 7.3)	
FPRH%	30.2%	29.9%	<0.001
	(SD 7.7)	(SD 7.2)	
Current smoker	226	4205	<0.001
	3.4%	12.0%	
Excess alcohol use (>3 units per day)	891	2185	<0.001
	13.6%	6.2%	
Current GC therapy	661	2415	<0.001
	10.0%	6.9%	

FPLH: Fat percentage at the left hip; FPRH: Fat percentage at the right hip; FPA: Fat percentage at the abdomen.

Our primary analysis demonstrated patients post-COVID 19 were less likely to have reported a frailty fracture after adjustment for known predictors of fractures. This was seen at all fracture sites [odds ratio (OR) 0.83, 95% CI 0.77–0.88] as well as non-hip (OR 0.78, 95% CI 0.73–0.83) and hip fracture (OR 0.74, 95% CI 0.62–0.88) sites specifically. This can be seen in Table 2.

**Table 2.** Odds of patient reporting any, non-hip and hip fracture after the COVID-19 pandemic

	All fractures	Non-hip	Hip
Unadjusted	OR 1.28 95% CI 1.21–1.35	OR 1.20 95% CI 1.14–1.27	OR 1.06 95% CI 0.95–1.21
Adjusted for: age; biological sex; BMI; current smoker; current alcohol excess; current GC therapy; rheumatoid arthritis; family history of a fracture; past history of a fracture; left femoral neck T-score	OR 0.83 95% CI 0.77–0.88	OR 0.78 95% CI 0.73–0.83	OR 0.74 95% CI 0.62–0.88

Logistic regression analysis looking at the relationship between all fractures, non-hip and hip fractures in patients referred to our scanner post-COVID-19 lockdown.

In our analysis of BMD changes post-COVID-19, multiple linear regression revealed that patients referred after the COVID-19 lockdown had a significantly lower left femoral neck T-score compared with those referred before the lockdown. Specifically, the coefficient for the post-COVID-19 variable was −0.23 (β = −0.23, SE = 0.02, 95% CI: −0.27 to−0.18, *P* < 0.001). This reduction was significant even after adjusting for variables such as age, gender, BMI, smoking status, alcohol use, glucocorticoid therapy, rheumatoid arthritis, personal history of fractures and family history of fractures. The full analysis can be seen in Table 3.

**Table 3.** Multiple linear regression modelling the relationship between patients referred after the COVID-19 pandemic and the left femoral neck T-score after adjustment

Variable	Coefficient (β)	Standard error	95% confidence interval	*P*-value
Post-COVID-19	−0.23	0.02	−0.27, −0.19	<0.001
Age at scan (years)	−0.01	0.00	−0.01, −0.00	<0.001
Gender	0.01	0.02	−0.02, 0.05	0.44
BMI	0.01	0.00	0.01, 0.01	<0.001
Current smoker	−0.10	0.02	−0.14, −0.06	<0.001
Current excess alcohol	0.13	0.03	0.08, 0.18	<0.001
Steroid therapy	0.17	0.02	0.13, 0.21	<0.001
Rheumatoid arthritis	−0.04	0.03	−0.09, 0.02	0.16
Personal history of fracture	−0.44	0.03	−0.50, −0.38	<0.001
Family history of a fracture	0.10	0.02	0.07, 0.13	<0.001

Next we analysed whether patients referred after the pandemic were more or less likely to have been diagnosed with osteoporosis, osteopenia or normal BMD after they had their DXA scan with us. We found that patients were more likely to have been diagnosed with osteoporosis (OR 1.49, 95% CI 1.40–1.59) but were less likely to have been diagnosed with osteopenia (OR 0.86, 95% CI 0.81–0.91) as well as normal BMD (OR 0.84, 95% CI 0.80–0.90). The full analysis can be seen in Table 4.

**Table 4.** Odds of patients being diagnosed with osteoporosis, osteopenia or normal BMD (via DEXA) in patients referred after the COVID-19 pandemic

	Normal BMD	Osteopenia	Osteoporosis
Unadjusted	OR 0.77 95% CI 0.73–0.82	OR 0.89 95% CI 0.84–0.93	OR 1.59 95% CI 1.50–1.69
Adjusted for: Age; biological sex; BMI; current smoker; current alcohol excess; current GC therapy; rheumatoid arthritis; family history of a fracture; past history of a fracture; left femoral neck T-score	OR 0.84 95% CI 0.80–0.90	OR 0.86 95% CI 0.81–0.91	OR 1.49 95% CI 1.40–1.59

Logistic regression analysis looking at the relationship between patient who were reported to be osteoporotic, osteopenic and normal BMD before versus after the COVID-19 lockdown.

## Discussion

This study is among the first to investigate how COVID-19 lockdowns has affected FRAX risk factors, BMD, and fragility fractures in an at-risk, referred population. While previous research has often focused on the acute effects of lockdowns on fractures [8–10], our study examines a larger sample of patients referred after the lockdown, up until February 2024. With access to bone density data, we were also able to assess how BMD has been impacted in these post-pandemic referrals. It’s important to note that our findings are specific to an at-risk, referred population, with data collected at a single point in time, without prospective or retrospective follow-up. Therefore, we caution against generalizing our results to the broader population but believe our research will generate new hypotheses and prompt further investigation.

In Table 1, we compared pre- and post-COVID-19 referred populations and found several statistically significant differences. Notably, post-COVID-19 patients exhibited lower BMD at both femurs and the lumbar spine, with the average patient diagnosed with osteopenia. These patients were also around 3 kg heavier, with higher adiposity levels and BMIs, compared with those referred before the pandemic.

Given the significantly lower BMD observed at all three sites analysed, we conducted an additional analysis utilizing a multiple linear regression to model the effect size while adjusting for confounders. We found that patients referred after the pandemic had a decrease of −0.23 units in their left femoral neck T-score, even after adjusting for known confounders. Moreover, as shown in Table 4, patients referred after the pandemic were more likely to be osteoporotic and less likely to be osteopenic or have normal BMD after they had their scan. This suggests that patients referred to our scanner post-pandemic had worse bone densities and a higher risk of being diagnosed with osteoporosis compared with patients prior to the pandemic.

To our knowledge, this is one of the first studies to suggest a potential association between COVID-19 lockdowns and reduced BMD in an at-risk, mostly elderly population. This finding aligns with previous research on bone density reduction in younger, healthy populations as a result of lockdown [11]. Although our study is cross-sectional, it is plausible that longitudinal studies may observe a similar association. We believe this could be due to reduced physical activity [12], dietary modifications [13], as well as an increase in risky behaviours such as excess alcohol consumption [14], all of which may have affected bone density. While some might argue that the worse BMD could be a result of patients not being scanned during the lockdown or changes in referral patterns and thus patients being older at the time of their scan, we believe the robustness of our analysis strengthens our results as we had adjusted for age and other confounders and still found worse bone density in post-pandemic referrals. Therefore, we believe patients may have worse bone densities now than before the pandemic; however, we recommend further research to confirm our hypothesis.

Interestingly, despite the higher likelihood of osteoporosis and lower average BMD in post-lockdown patients, this did not correspond to an increased fracture risk. In fact, we found that these patients were less likely to report a fracture including hip and non-hip, even after adjusting for known predictors. This is somewhat contradictory, as lower BMD is typically associated with an increased fracture risk [15]. One possible explanation is that patients who were typically at risk of experiencing fragility fractures instead experienced excess morbidity and mortality from the COVID-19 pandemic. Furthermore the relative reduction in physical activity due to the pandemic may have also conferred a protective effect as patients were less likely to fall and fracture. Hence, these multitude of factors may have lowered the odds seen in our populations as well as previously reported cohorts. Consequently, it could be argued that given we have returned to normality patients may now be at an increased risk of fracture due to the worse bone mineral density these patients are presenting with, however, this was not seen in our present study.

Ultimately, we recommend further research with prospective cohorts to investigate the temporal relationship between COVID-19 lockdowns, bone density, and fragility fracture risk. However, our findings suggest that clinicians should take proactive steps to mitigate these risks, including encouraging patients to build muscular strength and undertake weight bearing exercises, which has been shown to protect against fragility fractures and improve bone density. Additionally, clinicians should adopt a proactive screening approach, referring elderly individuals for DXA scans, as there may be many undiagnosed osteoporotic or osteopenic patients who could benefit from anti-resorptive therapy.

## Strengths and limitations

Our study has several limitations. Firstly, We acknowledge that our study is in a referred population and hence we are unable to extrapolate our findings to the general population. Furthermore, the impact of the pandemic did influence our referral patterns and hence this would have been an unadjusted source of bias present in our data. Another limitation of our study is that our referred population was rather homogenous consisting of a mainly Caucasian population further limiting our findings. One could also argue that information on dosages of glucocorticoids was also not included in our analysis; however, glucocorticoid use is also a binary variable in FRAX; hence, given our definition was the same this would have mitigated this source of bias.

Our study also has it strengths. Primarily the large number of patients referred post-COVID-19 plus the time frame as to which data was collected may have helped to limit any potential confounders in our analysis as well as allowing us the see true association. Furthermore, our patients underwent DXA which is the gold standard for bone density and limits measurement error. We also have worked within our study design by generating new hypotheses rather than making radical conclusions.

## Conclusion

In conclusion, our study revealed that, after adjusting for known confounders, patients referred after the COVID-19 pandemic exhibited significantly lower BMD compared with those referred before the pandemic, along with a higher likelihood of being diagnosed with osteoporosis. However, this did not correspond to an increased risk of fragility fractures; instead, there was a reduction in the odds of both hip and non-hip fractures amongst post-pandemic patients. This paradoxical finding may be explained by factors such as increased morbidity and mortality from COVID-19, as well as reduced physical activity during lockdowns which may have conferred an overall protective effect on fractures. Despite the current reduced odds of fracture, the observed decline in BMD could potentially increase fracture risk in these patients in the future. Further research is warranted to model temporal changes in fracture incidence and BMD in prospective cohorts to confirm and expand upon these findings.

## Data Availability

Data can be made available upon reasonable request to the corresponding author.
